# How different online recruitment methods impact on recruitment rates for the web-based *coortesnaweb* project: a randomised trial

**DOI:** 10.1186/s12874-019-0767-z

**Published:** 2019-06-19

**Authors:** Cauane Blumenberg, Ana Maria Baptista Menezes, Helen Gonçalves, Maria Cecília Formoso Assunção, Fernando César Wehrmeister, Aluísio J. D. Barros

**Affiliations:** Post-Graduate Program in Epidemiology, Federal University of Pelotas, 1160 Marechal Deodoro St. – 3rd floor – 96020-220, Pelotas, Brazil

**Keywords:** E-epidemiology, Epidemiology, Research subject recruitment, Survey methodology, Web surveys

## Abstract

**Background:**

The number of web-based E-epidemiologic studies using online recruitment methods is increasing. However, the optimal online recruitment method in terms of maximizing recruitment rates is still unknown. Our aim was to compare the recruitment rates of three online recruitment methods and to describe how these rates differ according to individual’s socioeconomic and demographic factors.

**Methods:**

A total of 2394 members of the 1993 Pelotas birth cohort that provided an e-mail address, a Facebook name, and a WhatsApp number during a face-to-face follow-up were randomly allocated to be recruited by e-mail, Facebook or WhatsApp (798 individuals per method). This was a parallel randomised trial applying a block randomisation (block size = 3). Between January and February 2018, we sent messages inviting them to register into the web-based *coortesnaweb* platform. Recruitment rates were calculated for each method, and stratified according to the individual’s socioeconomic and demographic characteristics. We also analysed absolute and relative inequalities on recruitment according to schooling and socioeconomic position.

**Results:**

Out of the 2394 individuals analysed, 642 registered into the platform. The overall recruitment rate was 26.8%. Recruitment rates for women were almost 10 percentage points higher compared to men. Facebook was the most effective recruitment method, as 30.6% of those invited through the social network were recruited. Recruitment rates of e-mail and WhatsApp were similar (recruitment rate = 24.9%). E-mail and Facebook were the most effective recruitment methods to invite highly educated and wealthier individuals. However, sending e-mails to recruit individuals also reflected in the highest inequalities according to schooling and socioeconomic position. In contrast, the lowest inequalities according to socioeconomic position were observed using Facebook.

**Conclusions:**

Facebook was the most effective online recruitment method, also achieving the most equitable sample in terms of schooling and socioeconomic position. The effectiveness of online recruitment methods depends on the characteristics of the sample. It is important to know the profile of the target sample in order to decide which online recruitment method to use.

**Trial registration:**

Brazilian Registry of Clinical Trials, identifier: RBR-3dv7gc, retrospectively registered in 10 April 2018.

**Electronic supplementary material:**

The online version of this article (10.1186/s12874-019-0767-z) contains supplementary material, which is available to authorized users.

## Background

The increase on the number of web-based E-epidemiologic studies is influenced by several factors, including the ever increasing number of people with access to broadband internet [1], the reduced study costs [2], and similar validity of the data compared to traditional data collection methods [3, 4]. A very important aspect to consider in web-based surveys is the way the participants are invited to participate, as the recruitment method can influence the recruitment rates (RECR) (proportion of all invited individuals that register to participate on the survey) [5–7], and consequently the response rates [6].

Some web-based epidemiologic studies compared online and offline recruitment methods, showing lower costs [5, 8, 9] and higher recruitment rates [5, 10] for the online methods. Additionally, using online recruitment methods is logistically simpler compared to offline methods, since it can be done by placing ads on websites or sending automatic messages over the internet [11, 12].

In the literature, it is described that recruitment rates of online methods can range from 6% to over 50% [5, 6, 13]. Until today, there is no optimum online recruitment method in terms of maximizing recruitment rates [9]. However, studies fail to formally compare the effectiveness of these methods. For this reason, the objective of this study was to compare the recruitment rates of three online recruitment methods in the context of the web-based *coortesnaweb* project.

## Methods

The 1993 Pelotas birth cohort is composed by 5249 participants who were born in that year from mothers living in the urban area of Pelotas, a medium-sized Southern Brazilian city. The original cohort sample was representative of all births occurring in the city [14]. In 2015, when the members of the birth cohort were 22 years of age, they were invited to participate in a face-to-face follow-up assessment, and 3810 individuals (76.3% of the original cohort, including those who died as being followed-up) were interviewed [15]. Several health-related and life-style subjects were investigated, including internet access and the use of online social networks. We asked the participants whether they had access to broadband internet, where and how often to quantify how many participants would be able to participate in a web-based project called *coortesnaweb*.

*Coortesnaweb* is an experimental web-based platform developed to collect epidemiological data from the members of the 1993 Pelotas birth cohort. The data is collected by means of self-administered online surveys, designed using REDCap [16], which were integrated to the *coortesnaweb* platform. In order to encourage the participation and to reduce attrition, gamification strategies were developed. After responding to the questionnaires, the participants earn points and these points are used to unlock personal results about their health (e.g. level of physical activity, internet addiction). Additionally, the participants can earn badges after achieving some goals.

To be eligible to the *coortesnaweb*, the members of the 1993 Pelotas birth cohort had to have: (i) participated in the 2015 follow-up, (ii) confirmed that they had internet access at home or in their mobile device, and (iii) responded to the 2015 face-to-face interview without help of a third individual. A total of 3537 cohort members (67.4% of the original cohort) were eligible to participate of the *coortesnaweb* project. However, since this study focuses only on the effectiveness of online recruitment methods, only the 2394 cohort members that provided an e-mail address, a Facebook name, and a WhatsApp number during the 2015 face-to-face assessment were considered eligible for this study.

This was a parallel randomised trial with equal groups. The 2394 eligible cohort members were randomly assigned by the study coordinator to be exclusively recruited by one of the three online recruitment methods: e-mail, Facebook message or WhatsApp message (Facebook Inc. 2018. Menlo Park, CA, United States). A block randomisation (block size = 3) was applied to guarantee equal sample sizes on each group (798 individuals per group). The randomisation was performed using a two-step computer algorithm written in Stata 15 (StataCorp. 2017. Stata Statistical Software: Release 15. College Station, TX: StataCorp LLC). First, participants were randomly assigned between the blocks. Then, in the second step, participants were randomly assigned within the block to receive one of the recruitment methods. Each participant received at most three invitations to register into the *coortesnaweb* platform, using the same recruitment method. The invitation messages were sent within a 10-day interval, between 11 January and 19 February 2018, on different times and days of the week. If the participant had registered into the platform before receiving the third invitation message, no more messages were sent. The invitation messages were tailored to each individual (using their first name), were written in Portuguese and had the same content, independent of the recruitment method used. The only exception was the e-mail message that also had a subject line, since the structure of e-mail messages require a subject (see Additional file 1).

E-mail messages were sent to the group allocated to be recruited by e-mail using mail merge, sending 300 e-mails per day to avoid spam filters. The WhatsApp numbers of the individuals allocated to be recruited by WhatsApp were added to the contact list of the *coortesnaweb* mobile phone, and the messages were sent manually. Facebook profiles of the individuals allocated to be recruited by Facebook were searched by two project members, and the invitation messages were also sent manually. All recruitment messages were sent by these two project members. The first out of the three rounds of invitation messages finished after 29 days of recruitment, however the number of days to complete the first round varied according to the recruitment method. All individuals assigned to the e-mail group were recruited after 8 days, while the first round of recruitment for the WhatsApp and Facebook groups took 13 and 28 days, respectively.

The main outcome analysed was the recruitment rate [17], which was computed as the ratio between the number of individuals who had registered and the total number of individuals invited (irrespective of a successful contact or not, since e-mails could return, WhatsApp numbers could be wrong and Facebook profiles could not be found). The recruitment rate for each group was calculated in order to compare their effectiveness.

The recruitment rate was also calculated according to sex, schooling, skin colour, and socioeconomic position (SEP). Skin colour was self-reported and categorized as white, brown, black, and other. This is commonly used as a proxy for social disparities in the Brazilian population [18]. Schooling was measured as total completed years of schooling until 2015, and categorized into 0–8, 9–11, and 12 or more years. Socioeconomic position was estimated through a wealth index based on the ownership of a set of assets (e.g. computers, vehicles, etc), on the characteristics of the household (e.g. number of bathrooms, number of rooms, etc) and on the education of the head of the household in 2015. It is considered a more stable and easier to collect socioeconomic measure compared to the income. The index was calculated using principal components analysis, and individuals divided into five equal sized groups (quintiles). More information about the index can be obtained elsewhere [19].

Relative and absolute inequalities in recruitment rate were calculated according to schooling and socioeconomic position using the concentration index (CIX) and the slope index of inequality (SII), respectively. The CIX is calculated by ranking the individuals according to groups of schooling or socioeconomic position in ascending order. If the CIX is zero, the recruitment rate would be equal across individuals, positive CIX values indicate higher recruitment rates among richer or better educated individuals, while negative values indicate higher recruitment rates among the poorer and less educated. The SII was calculated by a logistic regression, using the recruitment rate as outcome and the schooling or socioeconomic position as exposure variables. The slope index can be interpreted as the difference between the recruitment rates (expressed in percentage points) of the top and the bottom groups of schooling and socioeconomic position. Detailed information about these measures can be obtained elsewhere [20]. All the results presented in tables were obtained using intention to treat analyses. Per-protocol analyses, considering only the contacted individuals in the analyses, are provided in Additional file 3. The characteristics of the sample and the differences on recruitment rates were assessed using chi-squared test. All analyses were conducted using the Stata 15 (StataCorp. 2017. Stata Statistical Software: Release 15. College Station, TX: StataCorp LLC).

## Results

Compared to the cohort participants seen in 2015, at 22 years of age, the eligible participants for the *coortesnaweb* study were more educated, wealthier, and more likely to be white. There was no statistical difference according to sex (Table 1). After the randomisation process all groups were comparable according to sex, skin colour, schooling and socioeconomic position (Table 2).

**Table 1:** Comparison of the sample followed-up at 22 years of age and the group eligible to participate of the coortesnaweb and the recruitment study. Pelotas, Brazil, 2018

	Followed-up at 22 years of age		Eligible to participate of the *coortesnaweb*		Pearson’s chi-squared test		
	N	% (95% CI)	N	% (95% CI)	Value	DF	*P* value ^**a**^
Total	3810		2394				
Sex					3.0	1	0.084
Female	2027	53.2 (51.6, 54.8)	1310	54.7 (52.7, 56.7)			
Male	1783	46.8 (45.2, 48.4)	1084	45.3 (43.3, 47.3)			
Schooling (years)					602.6	2	< 0.001
0–8	1124	29.5 (28.1, 31.0)	355	14.9 (13.5, 16.3)			
9–11	1560	41.0 (39.4, 42.6)	1049	43.8 (41.9, 45.9)			
12+	1121	29.5 (28.0, 30.9)	987	41.3 (39.3, 43.3)			
Skin colour					56.1	3	< 0.001
White	2262	63.3 (61.7, 64.9)	1531	68.3 (66.4, 70.3)			
Brown	637	17.8 (16.6, 19.1)	338	15.1 (13.7, 16.6)			
Black	538	15.1 (13.9, 16.3)	288	12.9 (11.5, 14.3)			
Other	137	3.8 (3.3, 4.5)	82	3.7 (3.0, 4.5)			
Socioeconomic position					149.4	12	< 0.001
1st (poorest)	761	20.0 (18.8, 21.3)	310	13.0 (11.7, 14.4)			
2nd	761	20.0 (18.8, 21.3)	431	18.0 (16.5, 19.6)			
3rd	761	20.0 (18.8, 21.3)	477	20.0 (18.4, 21.6)			
4th	761	20.0 (18.8, 21.3)	552	23.1 (21.5, 24.8)			
5th (richest)	760	20.0 (18.7, 21.3)	619	25.9 (24.2, 27.7)			

*CI* confidence interval, *DF* degrees of freedom

^a^ *P* value for heterogeneity

**Table 2:** Characteristics of the sample according to randomisation group. Pelotas, Brazil, 2018

	E-mail		WhatsApp		Facebook	
	N	% (95% CI)	N	% (95% CI)	N	% (95% CI)
Total	798		798		798	
Sex						
Female	433	54.3 (50.8, 57.7)	451	56.5 (53.1, 59.9)	426	53.4 (49.9, 56.8)
Male	365	45.7 (42.3, 49.2)	347	43.5 (40.1, 47.0)	372	46.6 (43.2, 50.1)
Schooling (years)						
0–8	115	14.4 (12.1, 17.0)	106	13.3 (11.1, 15.9)	134	16.8 (14.4, 19.6)
9–11	337	42.2 (38.8, 45.7)	358	45.0 (41.5, 48.5)	354	44.4 (41.0, 47.9)
12+	346	43.4 (40.0, 46.8)	332	41.7 (38.3, 45.2)	309	38.8 (35.4, 42.2)
Skin colour						
White	521	69.7 (66.3, 72.8)	511	68.3 (64.9, 71.6)	499	67.1 (63.7, 70.4)
Brown	114	15.2 (12.8, 18.0)	118	15.8 (13.3, 18.6)	106	14.3 (11.9, 17.0)
Black	89	11.9 (9.8, 14.4)	89	11.9 (9.8, 14.4)	110	14.8 (12.4, 17.5)
Other	24	3.2 (2.2, 4.7)	30	4.0 (2.6, 5.4)	28	3.8 (2.6, 5.4)
Socioeconomic position						
1st (poorest)	101	12.7 (10.5, 15.2)	96	12.1 (10.0, 14.5)	113	14.2 (12.0, 16.8)
2nd	148	18.6 (16.0, 21.4)	145	18.2 (15.7, 21.0)	138	17.4 (14.9, 20.2)
3rd	152	19.1 (16.5, 21.9)	159	20.0 (17.3, 22.9)	166	20.9 (18.2, 23.9)
4th	178	22.3 (19.5, 25.3)	180	22.6 (19.8, 25.6)	194	24.4 (21.6, 27.5)
5th (richest)	219	27.4 (24.5, 30.6)	217	27.2 (24.3, 30.4)	183	23.1 (20.2, 26.1)

*CI* confidence interval

**Fig. 1:**
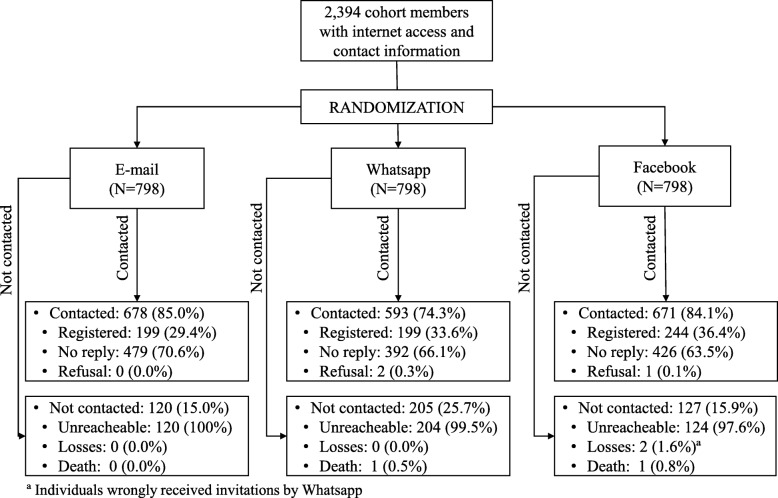
Flowchart depicting the logistic and design of the study, and the number of individuals contacted and not contacted by each recruitment method. Pelotas, Brazil, 2018

The flowchart presented in Fig. 1 shows that 85.0, 74.3 and 84.0% of the individuals assigned to be recruited by e-mail, WhatsApp and Facebook, respectively, could be contacted (e-mail did not return, WhatsApp number existed and Facebook profile could be found). There were three individuals that explicitly refused to participate, two from the WhatsApp and one from the Facebook group. There were two losses in the Facebook group, because they wrongly received invitations by WhatsApp. Two deaths were identified, one because a family member answered the WhatsApp message, and another because the Facebook profile was memorialized.

A total of 642 individuals registered into the platform, representing an overall recruitment rate of 26.8%. Of those, 491 individuals (76.5%) went on to complete the first questionnaire. Considering all the recruitment methods together, 31.0% (95% CI = 25.5, 33.6) of the invited females registered into the *coortesnaweb* platform compared to 21.8% (95% CI =19.4, 24.3) of the males.

**Fig. 2:**
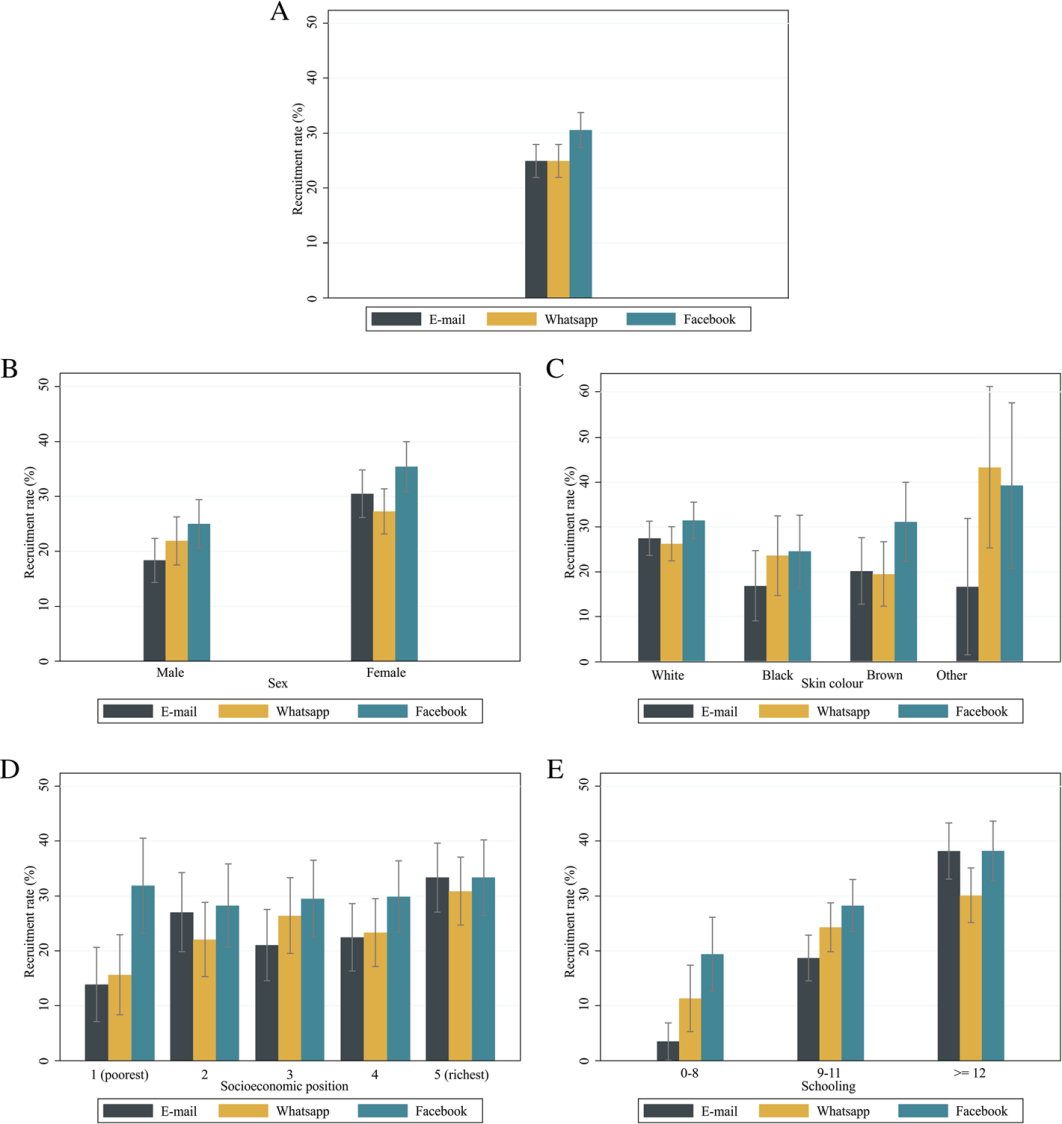
**a** Overall recruitment rate according to recruitment method and stratified by (**b**) sex, (**c**) skin colour, (**d**) socioeconomic position, and (**e**) schooling. Pelotas, Brazil, 2018

Figure 2A shows that the highest recruitment rate was obtained with Facebook (30.6%; 95% CI = 27.5, 33.9), followed by WhatsApp and e-mail (24.9%; 95% CI = 22.1, 28.1 for both methods), with a Chi-squared *p* value of 0.013. Sending Facebook messages was also the most effective method to recruit the poorest individuals (Fig. 2D). Moreover, it was seen that the recruitment rates obtained by using Facebook were similar across socioeconomic positions. In contrast, for e-mail and WhatsApp the recruitment rates increased with the increase of the socioeconomic position. E-mail, WhatsApp and Facebook methods achieved similar recruitment rates according to skin colour groups (Fig. 2C), and higher recruitment rates the higher the schooling (Fig. 2E). E-mail and Facebook recruitment rates were higher, compared to WhatsApp, to invite highly educated participants, with *p* value of 0.044. Detailed results including the percentages, confidence intervals and *p* values for each comparison are presented in Additional file 2.

The median number of days between the first invitation message and the day of registration considering only those who registered was also computed (data not shown in tables). Overall, it took a median of 4 days for participants to register into the *coortesnaweb* platform. The WhatsApp was the recruitment method that took the least time from the first invitation to registration, median of only 1 day (ranging from 0 to 31 days). For the Facebook method the median number of days was four (range: 0–45 days), while for e-mail it was six (range: 0–44 days).

The slope and concentration indexes presented in Table 3 showed that the highest inequalities on recruitment were observed in the e-mail group, for both schooling categories and socioeconomic position. The lowest inequalities according to socioeconomic position were observed in the Facebook group; being the slope index approximately a fifth compared to e-mail and WhatsApp methods. Overall, both absolute and relative inequalities on recruitment rates are higher according to schooling compared to the socioeconomic position.

**Table 3:** Overall inequalities on recruitment rates comparing schooling categories and socioeconomic position, and according to randomisation group. Pelotas, Brazil, 2018

	Overall		E-mail		WhatsApp		Facebook	
	SII (95% CI)	CIX (95% CI)	SII (95% CI)	CIX (95% CI)	SII (95% CI)	CIX (95% CI)	SII (95% CI)	CIX (95% CI)
Schooling	29.9 (23.8, 36.0)	9.3 (5.6, 13.0)	46.5 (37.1, 56.0)	24.4 (18.1, 30.7)	20.0 (9.4, 30.6)	1.7 (−5.1, 8.5)	24.5 (13.2, 35.7)	4.1 (−1.9, 10.0)
Socioeconomic position	11.3 (5.1, 17.4)	3.0 (−0.8, 6.8)	16.6 (6.2, 27.0)	8.7 (1.8, 15.6)	14.9 (4.6, 25.2)	4.7 (−2.2, 11.6)	3.1 (−8.1, 14.3)	−2.5 (−8.5, 3.6)

*CI* confidence interval, *CIX* concentration index, *SII* slope index of inequality

## Discussion

Our findings showed that using free online recruitment methods, we were able to recruit 26.8% of the eligible birth cohort members to participate in a web-based epidemiologic study. The most effective method was the Facebook. We also identified that e-mail and Facebook were the most effective methods to attract highly educated and wealthier individuals, and that the Facebook was the method that attracted the most equitable sample according to socioeconomic position. In our sample, the recruitment rate inequalities were higher according to schooling than according to socioeconomic position.

The overall recruitment rate of our web-based study was lower compared to the previous face-to-face follow-ups of the 1993 Pelotas birth cohort [15]. Two factors might explain these differences. First, in the face-to-face follow-ups we perform an extensive recruitment process, trying several times to contact the participant and using both online and offline recruitment methods, such as phone calls and home visiting. Second, in the face-to-face follow-ups we offer a monetary incentive for the participants, whereas in the web-based study we only offered non-monetary incentives (access to personal results by earning virtual points). Offering monetary incentives in web-based surveys can increase participation rates [21], but due to the lack of funding, we were not able to employ such approach.

Using Facebook ads to recruit participants to epidemiologic web-based studies is very common in the literature [5, 7, 9, 22–24]. However, when a study is not supposed to have open registration, but is focused on a list of eligible individuals, the use of Facebook ads may become very expensive and not feasible. We chose to send individual Facebook messages as we could confirm the eligibility of the individuals by checking their Facebook profile information (mainly using name, education, place and date of birth). Only one study, conducted in the United States, employed a similar approach as ours and achieved 24.6% of recruitment rate [13]. In our study, the Facebook recruitment rate was higher (30.6%).

The effectiveness of online recruitment methods depends on the characteristics of the sample [9]. Some studies state that sending e-mails is more effective to recruit older participants, and that Facebook would be more effective to recruit a sample of young adults [22, 25]. This may be one of the reasons why Facebook was the most effective recruitment method in our study, since individuals from 24 to 25 years of age composed our sample. Another reason that might explain the higher effectiveness of Facebook compared to the other recruitment methods is that errors in Facebook contact information provided during the face-to-face follow-up could be solved. Even if the participant had provided an incorrect Facebook profile name, in some cases we could identify the correct profile by searching the name of the individual into the social network and checking its personal information against the Facebook profile. In contrast, if there were errors in the WhatsApp number or in the e-mail address, it was not possible to identify or correct the error (apart from minor typing errors in the e-mail address).

To the best of our knowledge, this was the first study that used WhatsApp to recruit participants to an epidemiologic study. Although WhatsApp achieved a similar recruitment rate as sending e-mails, it was the method that presented the highest percentage of unreachable individuals – 25.6% versus around 15.0% for Facebook and e-mail. The higher percentage can be attributed to two factors: i) the impossibility to correct errors in WhatsApp numbers, and ii) the frequent changes in mobile phone numbers. In Brazil, the mobile carriers launch new plans that are usually financially better than the existing ones, encouraging clients to change plans and, sometimes, their phone number. If the unreachable individuals were not considered, the WhatsApp recruitment rate would reach 33.4% and would be similar to Facebook’s (see per-protocol analyses in Additional file 3).

Unlike WhatsApp, sending e-mails as recruitment method to epidemiologic research is more common in the literature. Similar to our finding, Buckingham and colleagues found e-mails to be less effective than recruitment via a social network [22]. In contrast, two other studies described that the recruitment rates of e-mails were higher than Facebook’s [7, 25]. The higher effectiveness of e-mails compared to Facebook can be attributed to the sample composition of these two studies, which were older than ours.

In our study, compared to Facebook, the recruitment via e-mail was related to higher absolute and relative inequalities according to schooling and socioeconomic position. A similar finding was described in a study that attracted better educated and richer participants using e-mails compared to Facebook recruitment [7]. Our hypothesis to explain this is that e-mails are mainly used for workplace and university communication, hence attracting individuals with formal jobs, with better education and from a higher socioeconomic position.

One notable finding is the marked sex differences on recruitment rates independent of the method used, which were almost 10 percentage points higher for females compared to males. This finding is consistent to three other web-based studies [7, 23, 25]. Another epidemiologic study, which also found higher participation among females, identified that the reasons for males not to participate are due to lack of interest and time constraints [26]. We did not investigate the reasons for not registering into the *coortesnaweb* platform, but we hypothesize that males could be less interested in the study as the participation of females was always higher than males’ in the previous face-to-face follow-ups of the 1993 Pelotas birth cohort [15].

A limitation that could have affected our study is the contamination between participants. For instance, a participant could have mentioned the study to another eligible individual before this individual read its recruitment message. This individual would become aware of the study by word of mouth but, in our analyses, he would be considered recruited by the method for which he/she was originally assigned. We could not quantify the total amount of contamination that could have affected our results, but two individuals assigned to receive e-mails were unreachable (did not receive any e-mail), but still registered into the coortesnaweb platform. Another limitation is related to the proportion of contacted individuals, which could be lower than estimated. This could happen if e-mail messages were redirected to spam folders, or if Facebook and WhatsApp messages were not read by the eligible individuals. We tried to avoid such issues by sending e-mails in small batches, and by trying to update the individual’s Facebook and WhatsApp information if the previous message was not read. As an alternative, we could use a mixed approach of recruitment methods, what could have increased the recruitment rates by decreasing the number of not contacted individuals. It is important to note that our findings are inserted in the context of the 1993 Pelotas birth cohort, a known sample that were already aware of the study and had participated of previous face-to-face follow-ups. In other situations, such as an unknown target population, other online recruitment methods could be employed (e.g. online ads) and their effectiveness could be different. Our study also presents some strengths: i) this was the first study that formally compared, using a randomised trial design, the effectiveness of online recruitment methods for epidemiologic research; ii) this was also the first web-based epidemiological study fully conducted online in the context of a middle-income country; and iii) we could compare the effectiveness of the recruitment methods using a standardized metric (the recruitment rate) [17], what usually does not happen in web-based epidemiologic research as the number of eligible individuals is not known.

## Conclusions

We were able to recruit members of a birth cohort to a web-based epidemiologic study using free online recruitment methods in the context of a middle-income country (Brazil). The effectiveness of the online recruitment methods is dependent on the individual characteristics of the target sample. Overall, the Facebook showed to be the most effective method to recruit young adults, also achieving the most equitable sample according to schooling and socioeconomic position. In contrast, the use of e-mails as a recruitment method might produce a biased sample in terms of socioeconomic factors. It is important to know the profile of the target sample in order to decide which online recruitment method to use.

## Additional files

Additional file 1: Invitation message content used in all recruitment methods, heading was only used in e-mail messages. Pelotas, Brazil, 2018. (DOCX 21 kb)

Additional file 2: Recruitment rate according to randomisation group stratified by individual characteristics using intention to treat analysis. Pelotas, Brazil, 2018. Detailed recruitment rate results to support the interpretation of the graphs displayed in Fig. 2. (DOCX 22 kb)

Additional file 3: Recruitment rate according to randomisation group stratified by individual characteristics using per-protocol analysis. Pelotas, Brazil, 2018. (DOCX 19 kb)

## Data Availability

The datasets used and/or analysed during the current study are available from the corresponding author on reasonable request.

## Abbreviations

CIX: Concentration index
RECR: Recruitment rate
SEP: Socioeconomic position
SII: Slope index of inequality
