# Supplementary material for: How different online recruitment methods impact on recruitment rates for the web-based coortesnaweb project: a randomised trial
Source: BMC Med Res Methodol. 2019 Jun 19;19:127. doi: 10.1186/s12874-019-0767-z (PMC6585038; doi:10.1186/s12874-019-0767-z)
Supplement: Supplementary file 1 — Invitation message content used in all recruitment methods, heading was only used in e-mail messages. Pelotas, Brazil, 2018. (DOCX 21 kb) [file 12874_2019_767_MOESM1_ESM.docx]

Additional file 1. Invitation message content used in all recruitment methods, heading was only used in e-mail messages. Pelotas, Brazil, 2018.

E-mail heading: Meet the new 1993 cohort platform on the Internet

Hello [name], how are you?

We would like to invite you to participate of the coortesnaweb.com, the new research platform of the 1993 Cohort on the Internet! In this platform you are able to respond to questionnaires, earn points and badges! With these points you will be able to unlock personal results about your health and compare you results to the other participants from the 1993 Cohort!

Register at coortesnaweb.com and start right now!

coortesnaweb team
