# Supplementary material for: How different online recruitment methods impact on recruitment rates for the web-based coortesnaweb project: a randomised trial
Source: BMC Med Res Methodol. 2019 Jun 19;19:127. doi: 10.1186/s12874-019-0767-z (PMC6585038; doi:10.1186/s12874-019-0767-z)
Supplement: Supplementary file 2 — Recruitment rate according to randomisation group stratified by individual characteristics using intention to treat analysis. Pelotas, Brazil, 2018. Detailed recruitment rate results to support the interpretation of the graphs displayed in Fig. 2. (DOCX 22 kb) [file 12874_2019_767_MOESM2_ESM.docx]

Additional file 2. Recruitment rate according to randomisation group stratified by individual characteristics using intention to treat analysis. Pelotas, Brazil, 2018.

|  | | **Overall** | | **E-mail** | | **Whatsapp** | | **Facebook** | | **P value ^a^** |
| --- | --- | --- | --- | --- | --- | --- | --- | --- | --- | --- |
|  | | **RECR** | | **RECR** | | **RECR** | | **RECR** | |  |
|  | | **N** | **% (95% CI)** | **N** | **% (95% CI)** | **N** | **% (95% CI)** | **N** | **% (95% CI)** |  |
| **Overall** | | 642 | 26.8 (25.1, 28.6) | 199 | 24.9 (22.1, 28.1) | 199 | 24.9 (22.1, 28.1) | 244 | 30.6 (27.5, 33.9) | 0.013 |
| **Sex** | |  |  |  |  |  |  |  |  |  |
| Female | | 406 | 31.0 (28.5, 33.6) | 132 | 30.5 (26.3, 35.0) | 123 | 27.3 (23.4, 31.6) | 151 | 35.5 (31.0, 40.1) | 0.031 |
| Male | | 236 | 21.8 (19.4, 24.3) | 67 | 18.4 (14.7, 22.7) | 76 | 21.9 (17.9, 26.6) | 93 | 25.0 (20.9, 29.7) | 0.092 |
| **Schooling (years)** | | |  |  |  |  |  |  |  |  |
| 0-8 | | 42 | 11.8 (8.9, 15.6) | 4 | 3.5 (1.3, 8.9) | 12 | 11.3 (6.5, 18.9) | 26 | 19.4 (13.5, 27.0) | 0.001 |
| 9-11 | | 250 | 23.8 (21.3, 26.5) | 63 | 18.7 (14.9, 23.2) | 87 | 24.3 (20.1, 29.0) | 100 | 28.3 (23.8, 33.2) | 0.013 |
| 12+ | | 350 | 35.5 (32.5, 38.5) | 132 | 38.2 (33.2, 43.4) | 100 | 30.1 (25.4, 35.3) | 118 | 38.2 (32.9, 43.7) | 0.044 |
| **Skin colour** | |  |  |  |  |  |  |  |  |  |
| White | | 434 | 28.4 (26.1, 30.7) | 143 | 27.5 (23.8, 31.5) | 134 | 26.2 (22.6, 30.2) | 157 | 31.5 (27.5, 35.7) | 0.155 |
| Brown | | 79 | 23.4 (19.2, 28.2) | 23 | 20.2 (13.8, 28.6) | 23 | 19.5 (13.3, 27.7) | 33 | 31.1 (23.0, 40.6) | 0.074 |
| Black | | 63 | 21.9 (17.5, 27.0) | 15 | 16.9 (10.4, 26.2) | 21 | 23.6 (15.9, 33.6) | 27 | 24.5 (17.4, 33.5) | 0.382 |
| Other | | 28 | 34.2 (24.7, 45.1) | 4 | 16.7 (6.3, 37.5) | 13 | 43.3 (26.8, 61.5) | 11 | 39.3 (23.0, 58.4) | 0.095 |
| **Socioeconomic position** | | | |  |  |  |  |  |  |  |
| 1st (poorest) | | 65 | 21.0 (17.9, 25.3) | 14 | 13.9 (8.4, 22.1) | 15 | 15.6 (9.6, 24.4) | 36 | 31.9 (23.9, 41.0) | 0.002 |
| 2nd | | 111 | 25.8 (21.6, 29.4) | 40 | 27.0 (20.5, 34.8) | 32 | 22.1 (16.0, 29.6) | 39 | 28.3 (21.4, 36.4) | 0.447 |
| 3rd | | 123 | 25.8 (23.2, 31.2) | 32 | 21.1 (15.3, 28.3) | 42 | 26.4 (20.1, 33.8) | 49 | 29.5 (23.1, 36.9) | 0.221 |
| 4th | | 140 | 25.4 (23.6, 31.6) | 40 | 22.5 (16.9, 29.2) | 42 | 23.3 (17.7, 30.1) | 58 | 29.9 (23.9, 36.7) | 0.194 |
| 5th (richest) | 201 | 32.5 (28.8, 37.3) | 73 | 33.3 (27.4, 39.9) | 67 | 30.9 (25.1, 37.4) | 61 | 33.3 (26.9, 40.5) | 0.824 |  |
| CI – confidence interval; RECR – recruitment rate  ^a^ Chi-squared test for heterogeneity | | | | | | | | |  |  |
