# Supplementary material for: How different online recruitment methods impact on recruitment rates for the web-based coortesnaweb project: a randomised trial
Source: BMC Med Res Methodol. 2019 Jun 19;19:127. doi: 10.1186/s12874-019-0767-z (PMC6585038; doi:10.1186/s12874-019-0767-z)
Supplement: Supplementary file 3 — Recruitment rate according to randomisation group stratified by individual characteristics using per-protocol analysis. Pelotas, Brazil, 2018. (DOCX 19 kb) [file 12874_2019_767_MOESM3_ESM.docx]

Additional file 3. Recruitment rate according to randomisation group stratified by individual characteristics using per-protocol analysis. Pelotas, Brazil, 2018.

|  | **Overall** | | **E-mail** | | **Whatsapp** | | **Facebook** | | **P value ^a^** |
| --- | --- | --- | --- | --- | --- | --- | --- | --- | --- |
|  | **RECR** | | **RECR** | | **RECR** | | **RECR** | |  |
|  | **N** | **% (95% CI)** | **N** | **% (95% CI)** | **N** | **% (95% CI)** | **N** | **% (95% CI)** |  |
| **Overall** | 639 | 32.9 (30.8, 35.0) | 197 | 29.1 (25.8, 32.7) | 198 | 33.4 (29.7, 37.3) | 244 | 36.1 (32.6, 39.9) | 0.022 |
| **Sex** |  |  |  |  |  |  |  |  |  |
| Female | 403 | 37.3 (34.4, 40.2) | 130 | 34.9 (30.2, 39.8) | 122 | 35.4 (30.5, 40.6) | 151 | 41.5 (36.5, 46.6) | 0.120 |
| Male | 236 | 27.4 (24.5, 30.5) | 67 | 22.1 (17.8, 27.1) | 76 | 30.6 (25.2, 36.7) | 93 | 29.9 (25.1, 35.2) | 0.038 |
| **Schooling (years)** | |  |  |  |  |  |  |  |  |
| 0-8 | 42 | 17.1 (12.9, 22.4) | 4 | 5.3 (2.0, 13.5) | 12 | 19.7 (11.5, 31.6) | 26 | 23.9 (16.7, 32.8) | 0.004 |
| 9-11 | 250 | 30.2 (27.1, 33.4) | 63 | 22.8 (18.2, 28.2) | 87 | 33.3 (27.9, 39.3) | 100 | 34.3 (29.0, 39.9) | 0.005 |
| 12+ | 347 | 40.0 (36.8, 43.3) | 130 | 40.0 (34.8, 45.4) | 99 | 36.7 (31.1, 42.6) | 118 | 43.2 (37.5, 49.2) | 0.296 |
| **Skin colour** |  |  |  |  |  |  |  |  |  |
| White | 431 | 33.6 (31.1, 36.3) | 141 | 30.7 (26.7, 35.1) | 133 | 34.0 (29.5, 38.9) | 157 | 36.4 (32.0, 41.1) | 0.194 |
| Brown | 79 | 31.2 (25.8, 37.2) | 23 | 25.3 (17.4, 35.2) | 23 | 29.1 (20.1, 40.1) | 33 | 39.8 (29.8, 50.7) | 0.107 |
| Black | 63 | 29.4 (23.7, 35.9) | 15 | 24.2 (15.1, 36.5) | 21 | 31.3 (21.3, 43.4) | 27 | 31.8 (22.7, 42.4) | 0.560 |
| Other | 28 | 40.0 (29.2, 51.9) | 4 | 19.1 (7.2, 41.8) | 13 | 59.1 (38.2, 77.2) | 11 | 40.7 (23.9, 60.1) | 0.027 |
| **Socioeconomic position** | |  |  |  |  |  |  |  |  |
| 1st (poorest) | 65 | 28.9 (23.3, 35.2) | 14 | 17.3 (10.5, 27.2) | 15 | 28.3 (17.8, 41.9) | 36 | 39.6 (30.0, 50.0) | 0.006 |
| 2nd | 111 | 33.0 (28.2, 38.3) | 40 | 32.8 (25.0, 41.6) | 32 | 32.3 (23.8, 42.2) | 39 | 33.9 (25.8, 43.1) | 0.967 |
| 3rd | 122 | 31.7 (27.2, 36.5) | 32 | 26.2 (19.2, 34.8) | 41 | 32.8 (25.1, 41.5) | 49 | 35.5 (27.9, 43.9) | 0.262 |
| 4th | 138 | 30.5 (26.5, 34.9) | 39 | 25.5 (19.2, 33.0) | 42 | 30.4 (23.3, 38.6) | 58 | 35.4 (28.4, 43.0) | 0.162 |
| 5th (richest) | 200 | 37.0 (33.1, 41.2) | 72 | 36.4 (29.9, 43.3) | 67 | 37.9 (31.0, 45.2) | 61 | 37.0 (29.9, 44.6) | 0.956 |
| CI – confidence interval; RECR – recruitment rate  ^a^ Chi-squared test for heterogeneity | | | | | | | | |  |
